# Billings F. on Three Recoveries from Tetanus

**Published:** 1888-09

**Authors:** F. Billings


### Billings F. On Three Recoveries from Tetanus.

The first recovery was that of a thirty-year-old man who had been wounded by a pitchfork through the foot, passing down from the dorsum. He did not come to the hospital until trismus had commenced. As the foot wound was suppurating, the house surgeon opened up the local wound, and dressed it antiseptically. Tetanus developed. The third day after the patient entered the hospital the sciatic nerve of that side was stretched without result. Three days afterward the crural nerves were stretched. Then the sciatic nerves was again stretched, without marked effect. At the same time the patient was given morphine and chloral. Afterward eighth grain hypodermic injections of physostigmine were then given every three hours until three grain doses were taken. The patient suddenly exhibited all the characteristic symptoms of physostigmine poisoning;—pin-point pupils, and rapid, weak pulse, œdema of the lungs with rapid superficial breathing. Under atropine and digitalis he soon recovered. The first thing he did was to assume the tetanus position—opisthotonos. He eventually recovered, but never regained his strength of mind. He was simple, had a peculiar laugh and talked a great deal. Before, he had been a silent man, but he became very garrulous, and was the clown of the ward.

A man was brought to the hospital with a simple fracture of one tibia. In two weeks trismus suddenly developed after a chill. On examining the man I detected fluctuation over the seat of fracture. The bones were freely movable. Dr. Isham was the attending surgeon. It was made a compound fracture and drained thoroughly. Although the trismus remained for several days, the man ultimately recovered, with perfect union of the bones.

A boy received a crushing injury of one leg. I amputated the leg in its upper third. The flaps united very well. About the seventh day he was taken with a chill, and trismus commenced, followed by tetanus. The friends of the boy consented to have the sciatic nerve stretched, but when we attempted it the warden and chairman of the hospital committee interfered, and commanded us to take the boy back. As a result the old medical board stepped out of the County Hospital. We afterward stretched the nerve and the boy recovered.

Dr. J. A. Robinson reports the case of a man who was brought in May, 1887, with what was supposed to be cerebral meningitis: he had a high temperature, face flushed and of a purple hue. He was easily excited, and in a day or two symptoms of tetanus were noticed, trismus and opisthotonos occurring during the progress of the case. During the January previous, he had received a wound from a buzz saw in the second and third fingers of the right hand. He was sent to the surgical side, and these fingers were amputated. For several days it was doubtful if he would recover. He finally did, but became insane.—*Medical Standard*, July, 1888.

Physical Education of the Blind.—A sound system of education has been developed by Dr. Campbell at the Royal Normal College for the Blind. A blind teacher gives instruction in singing, and connected with the college is a gymnasium, swimming bath, cycling track, and a boating lake. The College, says the *British Medical Journal*, is now educating 160 students, but it effects more than that for the public good in training a large number of teachers, who go thence to all parts of the world. A Royal Commission is now inquiring as to the educational needs of the blind, and the best means of providing them.

Dr. S. Sexton exhibited a New Portable Battery for the Storage of Electro-Motor Force, and a New Head Lantern for the Employment of Electric Light in Surgery.

The battery was made at his suggestion by the River and Rail Electric Light Co., of New York. It consists of three cells and will light a six-candle electric light. The lantern is a modification of that of Trouve, but much lighter and having a non-conducting base. Such a light is almost necessary in operations on the ear when ether is used as an anæsthetic. The battery will work continuously for twenty-four hours and will retain its power for several weeks or months. It may be charged by a dynamo or by twelve gravity cells. When not in use the storage battery may be kept in connection with the gravity cells.

Dr. S. Sexton: I have seen many cases in which irritation in the mouth has been the cause of naso-pharyngeal catarrh and aural symptoms. A lady was brought to me with intense pain in the ear and the head. There was nothing in the condition of the ear to account for these symptoms. Examination of the mouth showed that she was wearing a plate to bring the teeth closer together. The gum was intensely inflamed, although the patient complained of no discomfort. The removal of the plate caused a disappearance of the pain in the ear and head.

New Test for Bismuth.—To the Society of Chemical Industry Mr. F. B. Stone recently showed a very delicate test for bismuth. It depends on the fact that a strong solution of iodide of potassium produces a bright yellow color when added to a very dilute solution of sulphate of bismuth, containing only a small quantity of free sulphuric acid. One part of bismuth in 1,000,000 will show a distinct coloration.

Asphalte Pavements and the Public Health.—The vapor of tar has been supposed to be beneficial in a number of disorders, but Dr. Edmund J. Mills, of the Glasgow Technical College, has written a short note on the injurious effects of tar vapors so copiously discharged on our streets while asphalte road-mending is going on. It is said that the injurious effects of these fumes is perfectly well known at tar works, where the pitch is always cooled down in a closed chamber prior to casting in blocks. Casual inquiries have convinced him that the operations of road repair in Glasgow have been, during the last three weeks, the cause of a great deal of totally unnecessary illness, the leading symptoms of which are nausea and giddiness. He himself has been three times prostrated in this way, and has been thereby debarred from pursuing his ordinary professional work until these repairs cease. In view of the serious inconvenience from which many more must have suffered, it is to be hoped that the use of pitch in the future may be dispensed with, as the operation of road-mending can, if desired, be conducted without any offense whatever to the public health.—*British Medical Journal*, May 12, 1888.

